# The burden of the most common rheumatic disease in Colombia

**DOI:** 10.1186/s41927-021-00234-y

**Published:** 2022-01-20

**Authors:** Francy-Milena Cuervo, Ana M. Santos, John Londono, José-Ignacio Angarita, Juan C. Rueda, Rodrigo Giraldo-Bustos, Jesús Giovanny Ballesteros-Muñoz, Eugenia-Lucia Saldarriaga, Diana Padilla-Ortiz, Viviana Reyes-Martinez, Ingris Peláez-Ballestas, Diana Diaz-Jiménez, Pedro Santos-Moreno, Carlos E. Pinzón, Carlos Castañeda-Orjuela

**Affiliations:** 1 Grupo de Investigación Espondiloartropatías, Universidad de La Sabana – Hospital Militar Central, Bogotá, Colombia; 2 Rheumatology Unit, Hospital General de México, México City, Mexico; 3 National Health Observatory, National Health Institute, Bogotá, Colombia; 4 BIOMAB IPS, Bogotá, Colombia; 5 Epidemiology Department, Universidad de La Sabana, Chía, Colombia

**Keywords:** Burden of disease, Rheumatoid arthritis, Low back pain, Osteoarthritis, Colombia

## Abstract

**Background:**

Estimating the burden of rheumatic diseases (RDs) requires proper evaluation of its lethal and nonlethal consequences. In Colombia, it is possible to find local data and Global Burden of Disease (GBD) reports that collect information from varied contexts and apply complex statistical models, but no on-site estimations are available.

**Methods:**

This was a descriptive study on the burden of RD based on occurrence and mortality data in the general population during 2015, including information and prevalence estimations from the Community Oriented Program for the Control of Rheumatic Diseases (COPCORD) study. Disability-adjusted life years (DALYs) were estimated by combining measures of years of life lost (YLL) and years lived with disability (YLDs). For disability weight estimations among cases, different COPCORD responses were mapped using flowcharts to show the severity distribution according to GBD. All model parameters and results were validated through an expert consensus panel.

**Results:**

Low back pain (LBP) was the RD with the greatest burden of disease, costing 606.05 (95% CI 502.76–716.58) DALYs per 100,000 inhabitants, followed by osteoarthritis (292.11; 95% CI 205.76–386.85) and rheumatoid arthritis (192.46, 95% CI 109.7–239.69).

**Conclusions:**

The burden of RD is as high in Colombia as in other countries of the region. The results offer an interesting tool for optimizing healthcare system design as well as for planning the distribution of human and economic resources to achieve early diagnosis and adequate care of these diseases.

**Supplementary Information:**

The online version contains supplementary material available at 10.1186/s41927-021-00234-y.

## Background

Rheumatic diseases (RDs) are non-fatal chronic conditions that characteristically present with pain and physical limitations as well as overall functional dependence. They constitute an increasing problem that should not be underestimated [1]. Up to 50% of people with inflammatory diseases such as rheumatoid arthritis (RA) may develop permanent disability as a result within 4.5 to 22 years of the diagnosis [2]. Burden of disease (BoD) studies estimate the consequences of having a disease by measuring morbidity and mortality in the population [3]. Previous studies have shown that the burden of RD has been increasing globally since 2010 [4], and the same trend is anticipated to continue as the life expectancy of the population increases.

In 2019, the Global Burden of Disease (GBD) studies reported estimates identifying the most common conditions for varied regions of the world, including some RDs [5]. The researchers applied a robust methodological approach imputing missing information with meta-regression analyses. For example, in Colombia, they extrapolated data from regions that share common circumstances [5] to measure disability-adjusted life years (DALYs) [6]. Additionally, regional BoD studies have been carried out in Colombia with estimates of RD. The first study was conducted in 2005 and used the prevalence of RD in the Afro-Colombian population [7]. The second study took place in 2010 and extrapolated the prevalence of RD from Brazilian data [8]. There have been other attempts to assess the burden of RA using national data collected in 2005 [9], but none of them include data representative of the full population.

The Community Oriented Program for the Control of Rheumatic Diseases (COPCORD) strategy [10] was applied to assess the prevalence of RD in Colombia with a population sampling strategy [11]. That study included standardized questionnaires that evaluated quality of life, mechanical load and patient functionality. The most prevalent RD was non-specific musculoskeletal complaints (prevalence in 16.09% of patients aged 18 or older), followed by osteoarthritis (OA) (10.81%), appendicular regional pain syndrome (9.73%), mechanical low back pain (LBP) (7.24%), and RA (1.49%) [11]. The present study uses these newly available data on the prevalence of RD in addition to data found in secondary sources to estimate the burden of RD in DALYs for the Colombian population.

## Methods

### Study design

This is a descriptive analysis of the burden of RD in DALYs in the Colombian population using secondary information about the prevalence and mortality of LBP, OA and AR. A multiplicative model considering cases and deaths on the national scale was implemented to compute DALYs due to RD in Colombia. The parameters, model structure and estimations were validated by a panel of clinical experts.

### Subjects

The total number of cases included for each disease was taken from the study "Prevalence of rheumatic disease in Colombia, according to the COPCORD—Colombian Rheumatology Association strategy" [11]. This study used a validated questionnaire [12] and a primary care physician’s evaluation with an initial diagnosis; finally, experienced rheumatologists confirmed the initial diagnosis. Microdata from this populational survey were also input for the severity distribution. Causes of death were collected from death certificates, and it was noted if RD was identified as the basic cause of death.

#### Inclusion criteria

Participants above the age of 18 years who met the *American College of Rheumatology/European League Against Rheumatism* (ACR/EULAR) criteria for RA [13], knee [14] or hip [15] OA were included in our secondary analysis.

#### Exclusion criteria

All patients with inflammatory LBP who met the *European Spondyloarthropathy Study Group* (ESSG) criteria [16] or presented with any other LBP-associated diagnosis were excluded [17], according to GBD classification [6].

### Model parameters

#### Reference population

Data about the general population during 2015 were obtained through the National Statistics Administrative Department of Statistics (DANE; *Departamento Administrativo Nacional de Estadística*) and disaggregated by age and gender.

#### Prevalence and mortality of RDs

RD prevalence was obtained from the result of the COPCORD Colombia study, which included aggregate data for the population aged 18 years or older in six different Colombian cities during 2015, as well as disaggregated RD prevalence estimates by age and gender [11]. Data on mortality were obtained through DANE for each age group and sex for each RD during 2015. Diagnoses were defined using the International Classification of Diseases and Related Health Problems, tenth revision (ICD-10), for RD [6] (Table 1). Those involving cancer, trauma, infection, vascular disorders and inflammatory processes were excluded. Mortality rates according to sex were estimated. To estimate years of life lost (YLL), reference life table data (Additional file 1: Table S1) were obtained from the GBD 2019 study carried out by the Institute for Health Metrics and Evaluation (IHME) [5].

**Table 1:** Musculoskeletal disorders, equivalent ICD-10 codes and list of sequelae for each disorder in the GBD study

Disorder	ICD-10 CODES	Sequelae associated
Low back pain	M471, M478, M479, M480, M488, M489, M541, M544, M545	Mild, moderate, severe and most severe, without leg pain Mild, moderate, severe and most severe, with leg pain
Osteoarthritis of the knee and hip	M150, M154, M159, M169, M179, M198, M199	Mild, moderate and severe
Rheumatoid arthritis	M05-M06	Mild, moderate and severe

*ICD-10* International Statistical Classification of Diseases and Related Health Problems, tenth revision

#### Disease severity

To identify the associated disability weight (DW) and years lived with disability (YLD), disease severity was categorized as defined in the GBD study [6] (Table 2). For RD patient distribution percentages, the cross-walking method was applied from microdata of the Colombian COPCORD study. This method reclassifies the RD severity by collecting specific questions from the Health Assessment Questionnaire (HAQ) [18, 19] and the EuroQol 5-dimensional quality of life scale (EQ-5D) [20, 21] (Additional file 2: Table S2, Additional file 3: Table S3 and Additional file 4: Table S4). RD severity was calculated for all patients with RA, LBP, or OA. Disability weight values for the estimation of YLD were obtained from GBD 2019 [5] (Table 2).

**Table 2:** GBD 2020 sequelae, health state lay descriptions, and disability weights

Diseases	Sequelae	Health state lay description	Disability weight
Low back pain	Mild without leg pain	The person has mild back pain, which causes some difficulty dressing, standing, and lifting things	0.02 (0.011–0.035)
	Moderate without leg pain	The person has moderate back pain, which causes difficulty dressing, sitting, standing, walking, and lifting things	0.054 (0.035–0.079)
	Severe without leg pain	The person has severe back pain, which causes difficulty dressing, sitting, standing, walking, and lifting things. The person sleeps poorly and feels worried	0.272 (0.182–0.373)
	Most severe without leg pain	The person has constant back pain, which causes difficulty dressing, sitting, standing, walking, and lifting things. The person sleeps poorly, is worried, and has lost some enjoyment in life	0.372 (0.25–0.506)
	Mild with leg pain	The person has mild back pain, which causes some difficulty dressing, standing, and lifting things	0.02 (0.011–0.035)
	Moderate with leg pain	The person has moderate back pain, which causes difficulty dressing, sitting, standing, walking, and lifting things	0,054 (0.035–0.079)
	Severe with leg pain	The person has severe back and leg pain, which causes difficulty dressing, sitting, standing, walking, and lifting things. The person sleeps poorly and feels worried	0.325 (0.219–0.446)
	Most severe with leg pain	has constant back and leg pain, which causes difficulty The person dressing, sitting, standing, walking, and lifting things. The person sleeps poorly, is worried, and has lost some enjoyment in life	0.384 (0.256–0.518)
Osteoarthritis of the knee and hip	Mild	The person has pain in the leg, which causes some difficulty running, walking long distances, and getting up and down	0.023 (0.013–0.037)
	Moderate	The person has moderate pain in the leg, which makes the person limp, and causes some difficulty walking, standing, lifting and carrying heavy things, getting up and down and sleeping	0.079 (0.054–0.11)
	Severe	The person has severe pain in the leg, which makes the person limp and causes a lot of difficulty walking, standing, lifting and carrying heavy things, getting up and down, and sleeping	0.165 (0.112–0.232)
Rheumatoid arthritis	Mild	The person has moderate pain and stiffness in the arms and hands, which causes difficulty lifting, carrying, and holding things, and trouble sleeping because of the pain	0.117 (0.08–0.163)
	Moderate	The person has pain and deformity in most joints, causing difficulty moving around, getting up and down, and using the hands for lifting and carrying. The person often feels fatigue	0.317 (0.216–0.44)
	Severe	The person has severe, constant pain and deformity in most joints, causing difficulty moving around, getting up and down, eating, dressing, lifting, carrying and using the hands. The person often feels sadness, anxiety and extreme fatigue	0.581 (0.403–0.739)

Global burden of 369 diseases and injuries in 204 countries and territories, 1990–2019: a systematic analysis for the Global Burden of Disease Study 2019. Lancet (London, England). 2020;396(10258):1204–22. [5]

### Burden of disease estimation

A model describing the prevalence, mortality and disability of RD was built based on Colombian epidemiological data (Table 3). The number of DALYs was estimated as the sum of YLL and YLD. YLL was determined from the age of death and reference life table. To estimate YLDs, we combined the severity distribution of the included diseases and the corresponding DW values [22].

$${\text{DALY}} = {\text{YLL}} + {\text{YLD}}$$

**Table 3:** Prevalence, mortality and disability of rheumatic disease in Colombia, 2015

Colombian population, 2015^∞^	Male	Female				
18–29 years	5,092,439	4,899,545				
30–39 years	3,293,728	3,451,975				
40–49 years	2,760,734	3,002,471				
50–59 years	2,322,517	2,576,764				
60–69 years	1,422,161	1,613,617				
70–79 years	719,504	891,955				
80 + years	284,046	405,568				

Prevalence (%)*	Low back pain		Osteoarthritis		Rheumatoid arthritis	
	Male	Female	Male	Female	Male	Female
18–29	4.9 (4.8–4.9)	8.9 (8.8–8.9)	1.9 (0.7–5.4)	2.2 (1.0–4.5)	0.0	0.5 (0.1–2.2)
30–39	9.7 (9.6–9.7)	7.5 (7.4–7.5)	0.9 (0.2–4.2)	4.0 (2.2–7.1)	0.7 (0.1–4.7)	1.1 (0.3–3.3)
40–49	7.7 (7.6–7.7)	7.4 (7.4–7.5)	10.1 (3.9–23.7)	12.9 (9.7–17)	0.0	5.3 (3.2–8.7)
50–59	9.9 (9.8–9.9)	6.7 (6.6–6.7)	11.9 (7.8–17.9)	23.1 (19.3–27.4)	1.4 (0.4–4.8)	3.2 (1.8–5.5)
60–69	5.5 (5.5–5.6)	5.9 (5.9–6.0)	13.2 (9.1–18.7)	29.4 (24.5–34.8)	0.0	2.3 (1.1–4.8)
70–79	8.1 (7.9–8.3)	4.6 (4.5–4.7)	27.9 (20.7–36.5)	38.2 (31.9–44.9)	1.6 (0.4–6.4)	3.4 (1.6–7.3)
80 +	4.7 (4.6–4.8)	1.9 (1.9–2.0)	27.1 (14.8–44.3)	35.6 (25.7–46.9)	0.0	3.1 (0.8–11.7)

Mortality by age group (n)^∞^	Male	Female	Male	Female	Male	Female
18–29 years	0	0	0	0	0	0
30–39 years	0	0	0	0	0	0
40–49 years	0	0	0	0	0	0
50–59 years	0	0	0	0	0	0
60–69 years	3	0	3	2	0	0
70–79 years	0	2	9	13	0	0
80 + years	3	1	12	47	0	0
Disability (%)^¥^						
Mild	44		63		55	
Moderate	28		28		33	
Severe	17		9		12	
Most severe	11					

*Prevalence calculated for patients aged 18 years or older. Londoño J, Peláez-Ballestas I, Cuervo F, Angarita I, Giraldo R, Rueda JC, et al. Prevalencia de la enfermedad reumática en Colombia, según estrategia COPCORD-Asociación Colombiana de Reumatología. Estudio de prevalencia de enfermedad reumática en población colombiana mayor de 18 años. Revista Colombiana de Reumatología. 2018;25(4):245–56 [11].

^∞^ Data from National Statistics Administrative Department of Statistics (DANE; Departamento Administrativo Nacional de Estadística)

^¥^ Cross-walking method (Additional file 2: Table S2, Additional file 3: Table S3 and Additional file 4: Table S4)

where

$${\text{YLL}} = {\text{N}} \times {\text{L}}$$

N = number of deaths, L = standard life expectancy at age of death in years.

$${\text{YLD}} = {\text{P}} \times {\text{DW}}$$

P = prevalence in number of cases, DW = disability weight.

The model was built and programmed using MS Excel®, in accordance with transparency and usability criteria for external users, with the possibility of applying it for other RD scenarios.

### Sensitivity analysis

Uncertainty for all model parameters was expressed as 95% confidence intervals (CIs). A probabilistic sensitivity analysis was performed for each parameter using the Monte Carlo method, which ran 10,000 iterations of the model where each parameter had independent stochastic variation within its range of uncertainty. Finally, central tendency and confidence intervals were calculated for each final estimate by RD and by sex.

### Expert panel validation

Parameters such as the model design, structure and preliminary results of this investigation were identified and validated through expert panel consensus. Clinical and methodological experts were given a questionnaire specifically designed for the study, and afterwards, a meeting following the Delphi method was organized, where consensus between all parts was evaluated [23, 24].

## Results

### Number of cases and disease severity

#### Low back pain

**Fig. 1:**
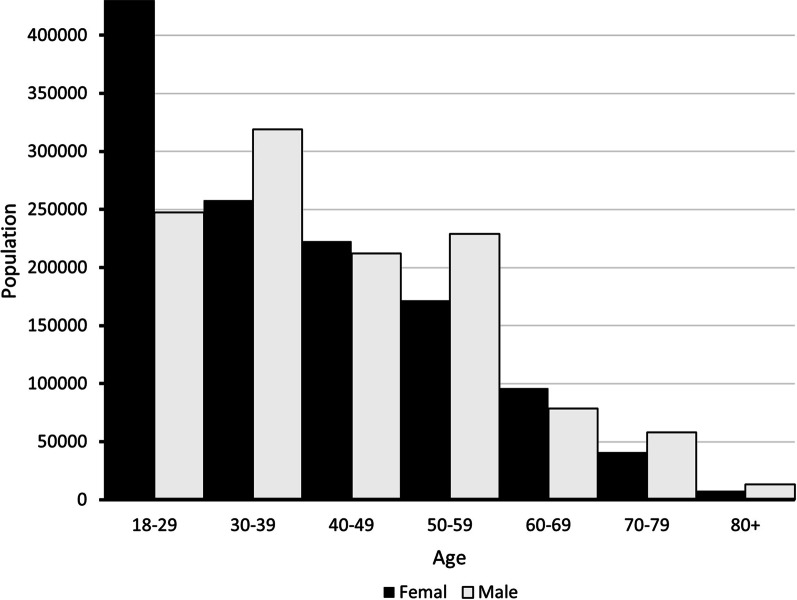
Age and sex distribution of low back pain cases

An estimated 2,390,256 cases were registered in 2015. Most of them were between the ages of 18 and 29 years, and more women than men were affected (Fig. 1). The severity distribution for low back with leg pain was 58% mild, 12% moderate, 12% severe, and 18% very severe. For low back without leg pain was 43% mild, 30% moderate, 18% severe, and 10 very severe %.

#### Osteoarthritis

**Fig. 2:**
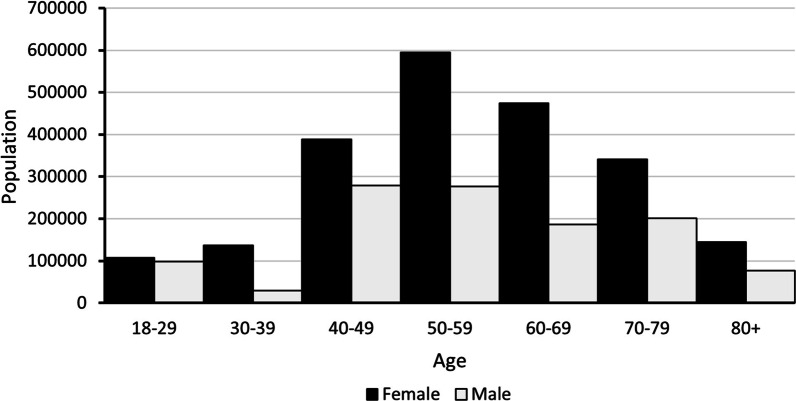
Age and sex distribution of osteoarthritis cases

This was the disease with most cases estimated by the model, with a total of 3,335,553 in 2015. Among them, 63% were mild, 28% were moderately severe, and 9% were very severe. Most patients were between 50 and 59 years of age (Fig. 2).

#### Rheumatoid arthritis

**Fig. 3:**
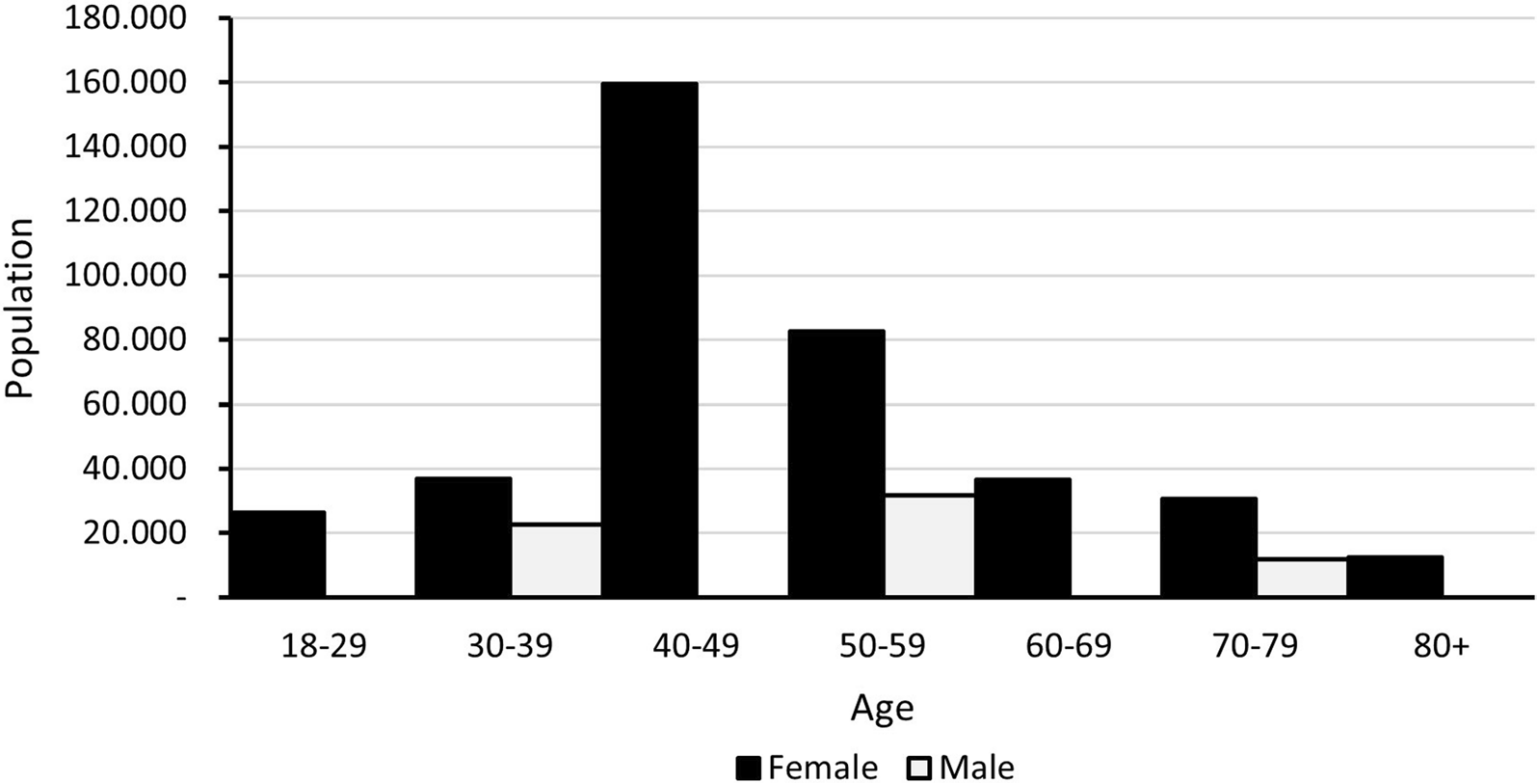
Age and sex distribution of rheumatoid arthritis cases

An estimated 451,173 cases were reported, mainly females aged between 40 and 60 years (Fig. 3). Regarding severity distribution, 55% of cases were mild, 33% were moderate and 12% were severe.

### Mortality

According to the ICD-10, there were no registered deaths attributable to RA in 2015. DANE was registered as a basic cause of death for 6 men and 3 women with LBP. For OA, 24 male deaths and 62 female deaths were recorded. Regarding the direct causes of death, one-quarter of deaths were related to complications of surgical procedures for spinal stenosis or OA. Approximately 45% of deaths were due to infections, and 25% were due to cardiovascular disorders such as pulmonary embolism and cardiovascular disease. In several cases, the direct cause could not be determined, possibly due to an error in the registry.

### Burden of rheumatic diseases in Colombia

The main source of RD burden in Colombia is LBP, followed by OA and RA. Table 4 represents years of healthy life lost for each of them. Table 5 corresponds to the BoD distribution in terms of DALYs per 100,000 population; most of the patients are female.

**Table 4:** Burden of disease in Colombia: low back pain, osteoarthritis and rheumatoid arthritis. Colombia, 2015

	Low back pain (95% CI)	Osteoarthritis (95% CI)	Rheumatoid arthritis (95% CI)
YLL*	62 (0–124)	393 (96–687)	0
YLD^¥^	198,341 (164,525 – 234,517)	95,237 (66,937–126,225)	63,007 (35,913 – 96,145)
DALY^∞^	198,403 (164,589 – 243,586)	95,629 (67,359–126,642)	63,007 (35,913–96,145)
DALYs per-100,000	606.05 (502.76 – 716.58)	292.11 (205.76–386.85)	192.46 (109.7–293.69)

*Years of life lost

^¥^Years lived with disability

^∞^Disability-adjusted life years

**Table 5:** DALY rates per 100,000 population by sex, Colombia, 2015

	Male	Female
Low back pain	96,051 (79,921–113,392)	102,352 (84,668–121,194)
Osteoarthritis	33,062 (21,388–46,830)	62,567 (45,971–79,812)
Rheumatoid arthritis	10,195 (1470–22,196)	52,812 (34,443–73,949)

#### Burden of disease model validation

The panel unanimously considered the estimation of total cases for LBP, OA, and RA for 2015 in Colombia to be reliable. As for the severity distribution data, the panel agreed with low back pain and OA findings but suggested RA flowchart redistribution to obtain more accurate results. The methodology and results for DALY calculation were approved. The panel determined that age had a stronger influence on LBP and OA than on RA. Considering the quality of information systems as well as the epidemiological profiles of RD, experts proposed Mexico, Brazil and Peru as adequate comparators of the results.

## Discussion

The present study is the first attempt to establish the burden of RD in Colombia by applying the methods used in GBD study using local data and data obtained through quality of life and functionality questionnaires. The burden of disease (BoD) for low back pain (LBP) was estimated to be 606.05 DALYs per 100,000 population, with a total of 2,390,256 cases. The estimates were 292.11 DALYs per 100,000 population, and a total of 3,335,553 cases of osteoarthritis (OA). There were 451,173 cases of rheumatoid arthritis (RA), with a DALY of 192.46 per 100,000.

Values for DALYs correspond mainly to YLD, which can be explained by the fact that the studied conditions tend to cause higher morbidity than mortality. The severity of disease is related to the estimation of YLD (Table 4). Patients who suffer from RDs experience severe limitations even in basic activities of daily living due to pain and fatigue [25].

LBP is a heterogeneous disease and a very common cause of outpatient consultations. Most patients require long-term conservative treatments [26]. Up to 25% of cases exhibit some disability when surgical intervention is required [27]. Overall, 32.4% of LBP patients present mild disability, 45.9% moderate, 20.7% severe, and 0.9% extreme [28] on the Oswestry Disability Index (ODI) [29]. Nonetheless, our analysis estimates more cases in the severe and extreme categories.

The prevalence of hip and knee OA increases past the age of 55 years, and it is likely to increase further due to the ageing of the population; thus, it is necessary to find cost-effective measures to mitigate long-term disability [30, 31] and to manage the resources of the Colombian healthcare system. The Western Ontario and McMaster Universities Osteoarthritis Index (WOMAC) [32], commonly employed to assess symptoms and functionality in patients, permits good radiological and functional correlation, allowing for a precise severity assessment. In one study, [33] the distribution of severity was as follows: 60.7% mild, 36% moderate and 3.3% severe. Similar results were found in this study.

Disease activity entails an increasing degree of disability in the natural course of RA [34]. Therefore, strict evaluation and treatment have a positive impact in reducing sequelae [35]. The disease severity distribution for the present study is similar to that found in other cohorts using the HAQ disability index: 63% mild, 25.2% moderate, and 11.8% severe [36].

The increase in population life expectancy affects the burden of RDs, as the prevalence and disability rates increase with ageing. In any case, it is important to consider the influence of other factors, such as tobacco use, inactivity, unhealthy diet and obesity, on the appearance of diseases at an early age [37].

### Comparison with local BoD estimates

DALY estimation for LBP corresponds to the GBD 2019 findings [rate of 951.74 DALYs per 100,000 population (95% CI 664.24–1275.07)]; for OA, the value was similar to that reported by the GBD 2019, with 226.45 DALYs per 100,000 population [95% CI 113.65–457.42)]. Meanwhile, DALY rates for RA were four times the value observed in the GBD study [51.26 DALYs per 100,000 population (95% CI 37.64–66.92)] [5].

RD prevalence was a key factor in determining our results because the values for RD prevalence in GBD 2019, 0.4% (95% CI 0.36–0.46) for RA, 11.41% (95% CI 10.11–12.77) for LBP and 8.73% (95% CI 7.84–9.70) for OA [5], were significantly lower than those used for the present study. Local RD prevalence data was sourced from a rigorously designed study [11].

There have been two previous BoD studies in Colombia. One registered burden of OA locally found it to be 380 DALY per 100,000 population in 2005 [7] and found this rate to increase up to 800 DALY per 100,000 population in 2010 for patients aged 70 to 79, including males and females [8]. In 2005, RA had 232 total DALYs per 100,000 population for women of all ages [7], and women aged 70 to 79 years, with 400 DALYs per 100,000 population, had the greatest value in 2010 [8]. Disability rates were the main contributor to DALY values in the 2005 and 2010 studies as well as in our own. It is worth noting that our results are lower than these values by half, and even though the methodology and data sources were different, we cannot discard the possibility of it being a direct result of treat-to-target strategies implemented locally [38].

When comparing our results to those from GBD 2019 [5] for countries with epidemiological characteristics similar to Colombia, we noted that the DALYs estimated for OA and RA were greater in the present study (Table 6). We suspect that factors such as delayed rheumatology consultation, limited access to second-line therapy, and the economic impact these imply for the healthcare system are potentially related to these findings [39].

**Table 6:** Disability-adjusted life years (DALYs) of low back pain, osteoarthritis and rheumatoid arthritis based on the GBD 2019 study

DALYs per 100,000*	Low back pain	Osteoarthritis	Rheumatoid arthritis
Mexico	738.45 (3520.76–985.55)	256.89 (131.12–499.99)	81.79 (63.01–104.47)
Peru	686.55 (483.68–923.38)	195.69 (98.42–395.25)	38.68 (28.09–51.01)
Brazil	943.44 (665.46–1253.4)	229.64 (115.24–454.73)	47.77 (34.77–61.59)

Global burden of 369 diseases and injuries in 204 countries and territories, 1990–2019: a systematic analysis for the Global Burden of Disease Study 2019. Lancet (London, England). 2020;396(10,258):1204–22. [5]. https://vizhub.healthdata.org/gbd-compare/

*DALYs per 100,000

### Comparison with other diseases

According to GBD 2019, cardiovascular disease is the main cause of DALY in Colombia [2819.8 DALY per 100,000 population (2257.73–3520.63)]. However, RD a highly prevalent group of diseases compared with other common conditions, such as diabetes [1000.6 (755.69–1282.64) DALY per 100,000 population], chronic obstructive pulmonary disease [596.18 (476.85–726.87) DALY per 100,000 population], chronic kidney disease [546.85 (444.28–671.65) DALY per 100,000], depression (307.03 (210.6–421.91) DALY per 100,000) and human immunodeficiency virus/acquired immunodeficiency syndrome [261.42 (246.72–277.29) DALY per 100,000 population] [5].

### Public health implications

The prevalence of osteoarticular disease is expected to progressively increase [40]. In the United States, a 2013 medical cost analysis found that half of all adult medical expenses were for arthritis patients. National medical costs attributable to arthritis were $139.8 billion (between $135.9 and $157.5 billion). It represents an additional $2117 per adult with arthritis [41]. Establishing healthcare models and therapies must be prioritized to guarantee timely diagnosis and adequate medical interventions in order to control disease activity and reduce disability progression [35], the activity of the disease is related to more medical costs. In addition, projections for the year 2030 estimate an increasing need for rheumatologists in the USA [42]. In Colombia, as in the United States, there is an uneven distribution of specialists, as they remain centralized in urban, densely populated areas [43]. The present study attempts to assess the current national RD status in an effort to evaluate the short-, medium-, and long-term impact these conditions have on healthcare systems.

It is important to recognize the limitations of this study. The first of these limitations is that the data originated from secondary sources and are therefore subject to possible registration bias. Because of this risk, all included data parameters were validated by an expert panel. Second, all prevalence data corresponded to highly populated areas, and no regional disaggregation was performed; instead, a national estimate was used. Another limitation of our study is related to the LBP classification as a rheumatic diseases. However, the classification used in our analysis is the same used by the GBD. These assessed diseases cannot yet be ungrouped, so it is very difficulty to collect good quality data on each topic.

Although it may not correspond to an inflammatory rheumatic disease, its frequency and impact on the functional capacity of affected individuals are very important.

## Conclusions

Rheumatic diseases in Colombia represent an important burden of disease in terms of DALYs. Our results could be considered a starting point for the design and development of public health policies and strategies to help mitigate disability and its effects on quality of life. The study was developed within the GBD framework because it is particularly useful in the analysis of rheumatic diseases and can easily be applied to study other rheumatic diseases not included in this study.

## Supplementary Information

**Additional file 1**. Institute for Health Metrics and Evaluation (IHME) and Global Burden of Disease Study 2016 (GBD 2016), reference life table.

**Additional file 2**. Cross walking algorithm for rheumatoid arthritis sequelae.

**Additional file 3**. Cross walking algorithm for low back pain sequelae.

**Additional file 4**. Cross walking algorithm for osteoarthritis sequelae.

## Data Availability

The datasets used and/or analysed during the current study are available from the corresponding author on reasonable request: johnlp@unisabana.edu.co.

## Abbreviations

BoD: Burden of disease
GBD: Global Burden of Disease
RD: Rheumatic disease
COPCORD: Community Oriented Program for the Control of Rheumatic Diseases
DALY: Disability-adjusted life years
YLLs: Years of life lost
YLDs: Years lost due to disability
LBP: Low back pain
RA: Rheumatoid arthritis
OA: Osteoarthritis
ACR/EULAR: American College of Rheumatology/European League Against Rheumatism
ESSG: European Spondyloarthropathy Study Group
DANE: Departamento Administrativo Nacional de Estadística
ICD: International Classification of Diseases
IHME: The Institute for Health Metrics and Evaluation
HAQ: Health Assessment Questionnaire
N: Number of deaths;
L: Standard life expectancy at age of death in years
P: Prevalence in number of cases
DW: Disability weight
ODI: Oswestry Disability Index
WOMAC: Western Ontario and McMaster Universities Osteoarthritis Index
EQ-5D: EuroQol five-dimensional questionnaire
