# Supplementary material for: The burden of the most common rheumatic disease in Colombia
Source: BMC Rheumatol. 2022 Jan 20;6:7. doi: 10.1186/s41927-021-00234-y (PMC8772222; doi:10.1186/s41927-021-00234-y)
Supplement: Supplementary file 1 — Additional file 1. Institute for Health Metrics and Evaluation (IHME) and Global Burden of Disease Study 2016 (GBD 2016), reference life table. [file 41927_2021_234_MOESM1_ESM.docx]

**Supplementary Table 1.** Institute for Health Metrics and Evaluation (IHME) and Global Burden of Disease Study 2016 (GBD 2016), reference life table

| **Age** | **qx** | **lx** | **Ex** |
| --- | --- | --- | --- |
| 0 | 0,001999 | 1 | 86,60328 |
| 1 | 0,000447 | 0,998002 | 85,7766 |
| 5 | 0,000328 | 0,997555 | 81,81428 |
| 10 | 0,000344 | 0,997228 | 76,84031 |
| 15 | 0,000739 | 0,996885 | 71,86577 |
| 20 | 0,000919 | 0,996149 | 66,91693 |
| 25 | 0,000949 | 0,995233 | 61,97612 |
| 30 | 0,001355 | 0,994289 | 57,0325 |
| 35 | 0,002064 | 0,992941 | 52,10627 |
| 40 | 0,003427 | 0,990892 | 47,20845 |
| 45 | 0,00574 | 0,987496 | 42,36145 |
| 50 | 0,008886 | 0,981828 | 37,59025 |
| 55 | 0,012829 | 0,973104 | 32,90342 |
| 60 | 0,019114 | 0,96062 | 28,29647 |
| 65 | 0,028786 | 0,942259 | 23,79594 |
| 70 | 0,048519 | 0,915135 | 19,42163 |
| 75 | 0,086647 | 0,870733 | 15,27302 |
| 80 | 0,163209 | 0,795287 | 11,46125 |
| 85 | 0,301613 | 0,665489 | 8,163226 |
| 90 | 0,501413 | 0,464769 | 5,531399 |
| 95 | 0,711972 | 0,231728 | 3,676063 |
| 100 | 0,867297 | 0,066744 | 2,623333 |
| 105 | 0,96231 | 0,008857 | 1,615458 |
| 110 | 1 | 0,000334 | 1,372479 |

**qx:** The probability that a person exact age x will die within one year.

**lx:** The number of persons surviving to exact age x.

**Ex:** The average number of years of life remaining at exact age x.
