# Supplementary material for: The burden of the most common rheumatic disease in Colombia
Source: BMC Rheumatol. 2022 Jan 20;6:7. doi: 10.1186/s41927-021-00234-y (PMC8772222; doi:10.1186/s41927-021-00234-y)
Supplement: Supplementary file 2 — Additional file 2. Cross walking algorithm for rheumatoid arthritis sequelae. [file 41927_2021_234_MOESM2_ESM.docx]

**Supplementary Table 2.** Cross walking algorithm for rheumatoid arthritis sequelae

|  | MILD \| | Dress ("without any difficulty” \| "with some difficulty”) & Reach ("without any difficulty” \| "with some difficulty”) & Stiffness ("without any difficulty” \| "with some difficulty”) & Fatigue ("without any difficulty” \| "with some difficulty”) & Stand up ("without any difficulty” \| "with some difficulty”) & Anxiety "no problems" |
| --- | --- | --- |
|  |  |  |
| RHEUMATOID ARTHRITIS | MODERATE \| | Dress ("without any difficulty” \| "with some difficulty” \| "with much difficulty”) & Reach ("without any difficulty” \| "with some difficulty” \| "with much difficulty”) & Stiffness ("without any difficulty” \| "with some difficulty” \| "with much difficulty”) & Fatigue ("without any difficulty” \| "with some difficulty” \| "with much difficulty”) & Stand up ("without any difficulty” \| "with some difficulty” \| "with much difficulty”) & Anxiety ("no problems” \| "moderately”) |
|  |  |  |
|  | SEVERE \| | Dress "unable to do” \| Reach "unable to do” \| Stiffness "unable to do” \| Fatigue "unable to do” \| Stand up "unable to do” \| Anxiety "extremely" |

| DRESS | Dress yourself, including shoelaces and buttons? | | |
| --- | --- | --- | --- |
| REACH | Reach and get down a 5-pound object (such as a bag of sugar) from above your head? | | |
| STIFFNESS | Lift a full cup or glass to your mouth? | | |
| FATIGUE | Walk outdoors on flat ground? | | |
| STAND UP | Stand up from a straight chair? | | |
| ANXIETY | Anxious or depressed |  |  |
