# Supplementary material for: The burden of the most common rheumatic disease in Colombia
Source: BMC Rheumatol. 2022 Jan 20;6:7. doi: 10.1186/s41927-021-00234-y (PMC8772222; doi:10.1186/s41927-021-00234-y)
Supplement: Supplementary file 3 — Additional file 3. Cross walking algorithm for low back pain sequelae. [file 41927_2021_234_MOESM3_ESM.docx]

**Supplementary Table 3.** Cross walking algorithm for low back pain sequelae

|  |  |  |
| --- | --- | --- |
|  | MILD | Dress "without any difficulty" & Stand up "without any difficulty" & Reach "without any difficulty" & Anxiety "no problems" |
|  |  |  |
|  |  | Stand up ("without any difficulty" \| "with some difficulty") & Walk ("without any difficulty" \| "with some difficulty") & Dress ("without any difficulty" \| "with some difficulty") & Reach ("without any difficulty" \| "with some difficulty") & Anxiety "no problems") |
|  | MODERATE \| |  |
|  |  | (Dress "with some difficulty" \| Reach "with some difficulty") & Stand up "with some difficulty" & Walk ("without any difficulty" \| "with some difficulty") & Anxiety "no problems" |
| LOW BACK PAIN |  |  |
|  | SEVERE | Reach ("without any difficulty” \| "with some difficulty” \| "with much difficulty”) & Dress ("without any difficulty” \| "with some difficulty” \| "with much difficulty”) & Anxiety ("no problems" \| "moderately") & (Stand up "without any difficulty” \| "with some difficulty”) \| Walk ("without any difficulty” \| "with some difficulty”)) |
|  |  |  |
|  |  | Reach ("with much difficulty " \| "unable to do") \| Anxiety ("no problems" \| "moderately" \| "extremely") \| Walk ("with much difficulty " \| "unable to do") \| Dress ("with much difficulty " \| "unable to do") \| Stand up ("with much difficulty " \| "unable to do")) |
|  | MOST SEVERE \| |  |
|  |  | Anxiety ("moderately" \| "extremely") & (Dress ("with some difficulty” \| "with much difficulty” \| "unable to do") \| Stand up ("with some difficulty” \| "with much difficulty” \| "unable to do") \| Reach ("with some difficulty” \| "with much difficulty” \| "unable to do") \| Walk ("with some difficulty” \| "with much difficulty” \| "unable to do")) |

| DRESS | Dress yourself, including shoelaces and buttons? | REACH | Bend down to pick up clothing from the floor? |
| --- | --- | --- | --- |
| STAND UP | Stand up from a straight chair? | ANXIETY | Anxious or depressed |
| WALK | Walk outdoors on flat ground? |  |  |
