# Supplementary material for: The burden of the most common rheumatic disease in Colombia
Source: BMC Rheumatol. 2022 Jan 20;6:7. doi: 10.1186/s41927-021-00234-y (PMC8772222; doi:10.1186/s41927-021-00234-y)
Supplement: Supplementary file 4 — Additional file 4. Cross walking algorithm for osteoarthritis sequelae. [file 41927_2021_234_MOESM4_ESM.docx]

**Supplementary Table 4.** Cross walking algorithm for osteoarthritis sequelae

|  | MILD \| | Stand up ("without any difficulty" \| "with some difficulty") & Arising ("without any difficulty" \| "with some difficulty") & Walk ("without any difficulty" \| "with some difficulty") & Up and down ("without any difficulty" \| "with some difficulty") & Reach ("without any difficulty" \| "with some difficulty") |
| --- | --- | --- |
|  |  |  |
| OSTEOARTHRITIS | MODERATE \| | Stand up ("without any difficulty” \| "with some difficulty” \| "with much difficulty”) & Arising ("without any difficulty” \| "with some difficulty” \| "with much difficulty”) & Walk ("without any difficulty” \| "with some difficulty” \| "with much difficulty”) & Up and down ("without any difficulty” \| "with some difficulty” \| "with much difficulty”) & Reach ("without any difficulty” \| "with some difficulty” \| "with much difficulty”) |
|  |  |  |
|  | SEVERE & | Stand up ("without any difficulty” \| "with some difficulty” \| "with much difficulty”\| "unable to do") & Arising ("without any difficulty” \| "with some difficulty” \| "with much difficulty”\| "unable to do") & Walk ("without any difficulty” \| "with some difficulty” \| "with much difficulty”\| "unable to do") & Up and down ("without any difficulty” \| "with some difficulty” \| "with much difficulty”\| "unable to do") & Reach ("without any difficulty” \| "with some difficulty” \| "with much difficulty”\| "unable to do") |

| STAND UP | Stand up from a straight chair? |
| --- | --- |
| ARISING | Get in and out of bed? |
| WALK | Walk outdoors on flat ground? |
| UP AND DOWN | Climb up five steps? |
| REACH | Reach and get down a 5-pound object (such as a bag of sugar) from above your head? |
